# A 3D-Printed Crown Integrated with 3D-Printed Orthodontic Brackets: A Novel One-Unit Printing Technique

**DOI:** 10.3390/ma18122727

**Published:** 2025-06-10

**Authors:** Suliman Y. Shahin, Essam A. Nassar, Mohammed M. Gad

**Affiliations:** 1 Department of Preventive Dental Sciences, College of Dentistry, Imam Abdulrahman Bin Faisal University, P.O. Box 1982, Dammam 31441, Saudi Arabia; sshahin@iau.edu.sa (S.Y.S.); enassar@iau.edu.sa (E.A.N.); 2 Department of Substitutive Dental Sciences, College of Dentistry, Imam Abdulrahman Bin Faisal University, P.O. Box 1982, Dammam 31441, Saudi Arabia

**Keywords:** 3D printing, bond strength, orthodontics, brackets

## Abstract

This study aimed to present a new application of 3D printing technology for crowns integrated with orthodontic brackets as one unit and to assess the strength of the bonded groups and the one-unit printed group. A total of 60 lateral incisors with brackets were obtained and allocated into two main groups: bonded groups and one-unit group. For the bonded groups, there were 40 specimens (20 conventionally fabricated crowns and 20 3D-printed crowns with bonded brackets), while for the one-unit group, there were 20 3D-printed crowns and brackets fabricated as one unit. One lateral incisor and one with a bracket were scanned, forming STL files for designing and printing 3D-printed specimens (20 without, 20 with brackets). Half of the specimens (30, *n* = 10) were thermocycled (5000 cycles). A universal testing machine was used for the bond strength (MPa) measurement, followed by analysis of the debonded areas and failure mode (adhesive, cohesive, or mixed). ANOVA and the post hoc Tukey’s test were used for analysis of the collected data (α = 0.05). The 3D-printed one-unit group significantly showed high strength compared with the bonded brackets (*p* < 0.001). The 3D-printed bracket showed the highest SBS (10.14 ± 1.93 MPa). After thermocycling, the bond strength of the bonded brackets significantly decreased (*p* < 0.001). The adhesive failure was dominant in the bonded groups, while the one-unit group exhibited all the fractures in the brackets. The introduced technique for producing a one-unit 3D-printed provisional crown integrated with orthodontic brackets is considered a clinically plausible option in contemporary orthodontic practice. However, further investigations are recommended to verify the findings of the present study before clinical implementation.

## 1. Introduction

Orthodontic treatment is frequently combined with other dental specializations like periodontics and prosthodontics [1,2]. It is also common to see patients with temporary crowns seeking orthodontic treatment to correct gaps in their front teeth due to missing teeth [3]. In the pre-restorative orthodontic treatment phase, orthodontists attach orthodontic brackets not only to natural teeth but also to teeth that have significant composite resin restorations or are covered with ceramic or temporary resin crowns [4]. In certain orthodontic treatments, tooth movement is required, but the crown cannot be used due to crown defects such as extensive carious lesions or fracture of the crown [1,4]. In these situations, teeth must be temporarily restored with a provisional crown before being extruded, aiming to maintain the biological width of the permanent crowns [5]. It provides optimum protection, stabilization, and functionality of the tooth, impacting the cosmetic outcome of the eventual restoration [5,6].

The success of orthodontic treatment and its timely completion largely depend on the bond strength at the bracket/tooth interface [1,5]. Research indicates that shear bond strength in the range from 6 to 8 MPa is optimal for orthodontic tooth movement [5,7]. In clinical settings, orthodontists are particularly focused on the shear bond strength between bracket adhesives and restorative surfaces. Poor bonding of the brackets to the temporary materials can result in a high rate of failure, negatively impacting the cost and effectiveness of orthodontic treatment, as well as patient comfort [1,7,8].

Earlier, Krey et al. proved the possibility of printing orthodontic brackets and reported the possibility of a treatment sequence when using a 3D-printing system for individualized printed brackets [9]. Recent advances in the computer-aided design software (3D orthodontics software, UBrackets, Deltaface (https://deltaface.shop/?utm_source=chatgpt.com), Avenue des Bénédictins, Limoges, France) empowered orthodontists to design and print customized orthodontic brackets directly within their office in the orthodontic clinic. The design workflow adheres to a structured protocol, which leads to easy and fast production [10]. One advantage of this technology for 3D-printed brackets is the sufficient strength required for tooth movement [11,12]. However, it showed a color change when immersed in the dietary solution [13]. The bracket base morphology can be influenced by various factors, including the size, retention form, and surface treatment, which are determined during the manufacturing and customization process [14,15]. Hodecker et al. suggested different designs of 3D-printed brackets (a macro-retentive and/or base enlargement) and reported the enhanced bond strength of the printed brackets through the implementation of a design featuring macro-retentive features and enlarged bases [15].

With advanced technology used in dentistry, 3D printing is on the rise in orthodontics, including producing in-office printed brackets [9,16,17,18]. The concept of resin-based printed brackets was recently proved and also bonded with 3D-printed provisional and permanent crown [19], allowing easy matching to the tooth color with the esthetics concerns of printed polymer brackets, making it a suitable alternative to metallic ones [20]. Moreover the printed brackets exhibited low sliding resistance [21,22,23] but showed less effective torque compared with ceramic and metal brackets [20,24,25]. Many attempts have been made to improve the strength of polymer-based printed brackets, such as using a novel permanent 3D-printed material with high strength. In terms of the accuracy of in-office-manufactured resin brackets, Bauer et al. [19] found that the slots of printed resin brackets have comparable accuracy to those of other bracket materials. In addition to the strength of printed brackets, the bond strength of printed brackets with a printable crown was reported by Çokakoğlu et al. [26]. In resin-to-bracket bonding, orthodontists often face the dual challenge of ensuring sufficient adhesion throughout treatment while also maintaining the structural integrity of the restorative material [27,28]. However, provisional dental prostheses provide challenges for orthodontists when it comes to adhesive failure [27,29], confirming that the bond strength at the bracket/resin interface is the point of weakness.

To harness the advantages of printing technology in the fabrication of crowns and brackets, a novel technique was introduced to address the bonding challenges and mitigate the risk of debonding. The single unit (bracket and crown) was designed and printed as one unit, which might be considered a way out of such a bonding problem and achieve long-term successful treatment. Therefore, this study sought to introduce and implement a new technique using additive technology for temporary restorations (3D-printed restoration with brackets as one unit) to improve the strength and avoid the debonding problems in conventional treatment. The null hypothesis was that a one-unit crown/bracket does not have a significant effect on the strength in comparison to traditional methods.

## 2. Materials and Methods

### 2.1. Sample Size and Specimen Grouping

**Figure 1:**
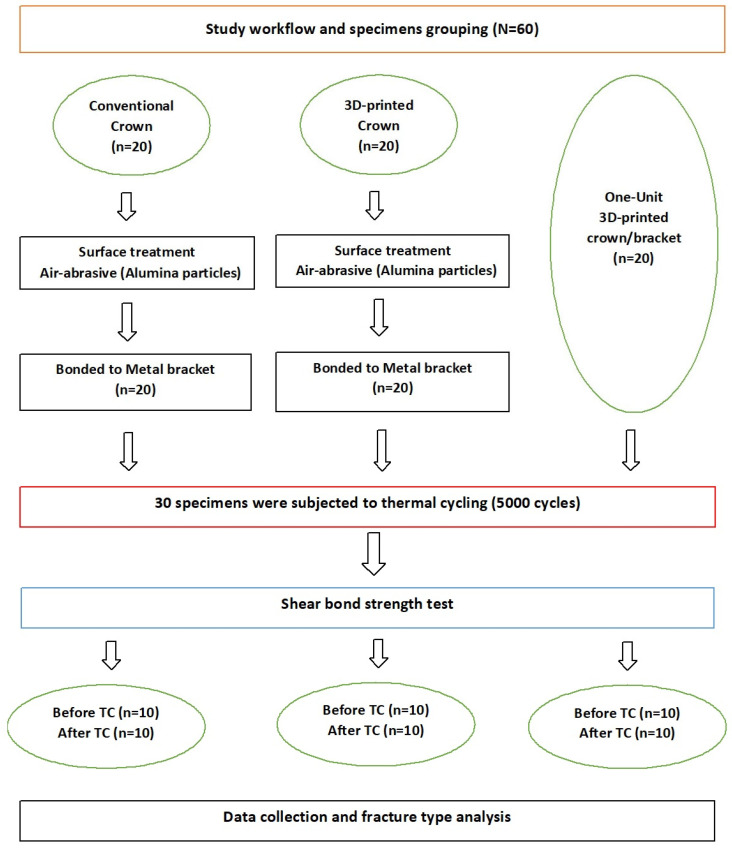
Study workflow.

The required sample size was determined using an online sample size calculator, referring to data from a previous study [30]. The calculation was performed with a significance level of α = 0.05, with a power of 0.80 and a 95% confidence level. Based on the sample size calculation, a total of 60 specimens were required. The specimens were divided into two bonded groups and a one-unit group. The bonded groups contained two groups according to crown fabrication technique: conventional bonded with metal bracket (*n* = 20), 3D-printed crown bonded with metal bracket (*n* = 20), and 3D-printed one-unit group (*n* = 20) (Figure 1). As shown in Figure 1, additional teeth were scanned and printed to prove the applicability of the introduced technique for anterior and posterior teeth.

### 2.2. Conventional Specimen Fabrication

The conventional provisional crown preparation was standardized by a customized mold for the lateral incisor. The mold was filled with the conventional provisional resin (Protemp™ Crown Temporization Material; Success CD, Neumünster, Germany), followed by a curing process that was completed according to the manufacturer’s recommendation, fabricating 20 crowns. A clear template was created to ensure consistent and uniform placement of the brackets on the prepared teeth. The metal bracket (3M^TM^ Unitek^TM^, Gemini Metal Brackets, 3M, St. Paul, MN, USA) and composite resins (Transbond^TM^ XT; 3M Unitek, Monrovia, CA, USA) were bonded on the labial surface of the fabricated teeth.

### 2.3. Three Dimensional-Printed Teeth Fabrication

**Figure 2:**
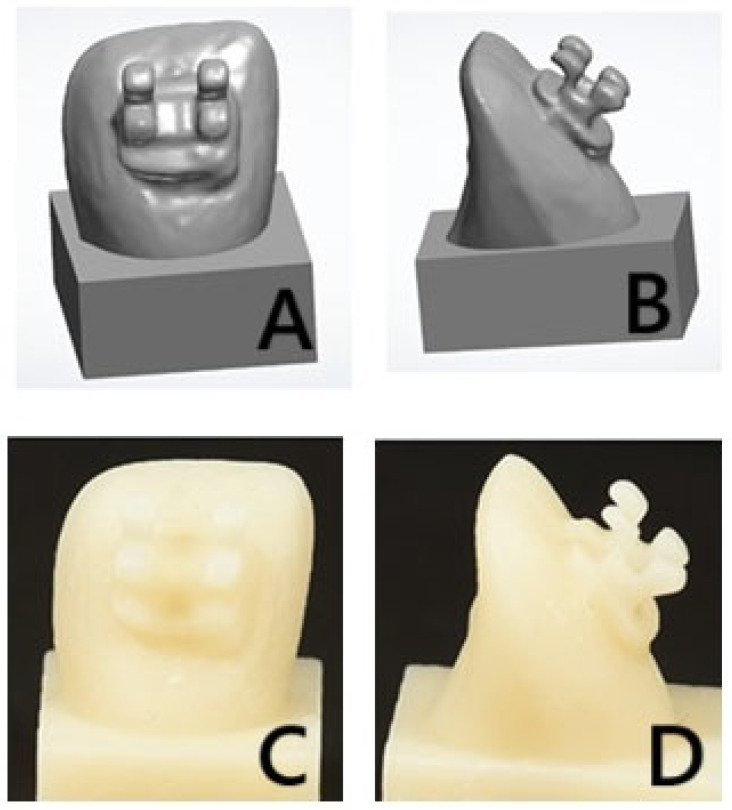
STL images of the designed specimen, (**A**) facial view and (**B**) proximal view, and printed specimens with brackets, (**C**) facial view and (**D**) proximal view.

A laboratory scanner (3Shape desktop scanner (3Shape A/S, Copenhagen, Denmark)) was used to scan two lateral incisors (one without and one with a bracket). After scanning, the STL file was imported was imported to the printer (NextDent^TM^ 5100, 3D Systems, Vertex Dental B.V., Soesterberg, The Netherlands) (Figure 2A,B), followed by slicing and supporting the structure configuration. The file was sliced and designed to be printed with the following parameters: 0-degree printing angle and 50 µm printing layer thickness. The specimens were cleansed using 99.9% isopropyl alcohol to remove any surface residues after printing. Afterwards, the specimens were subjected to additional polymerization in a post-polymerization unit (NextDent^TM^, LC-D Print Box, 3D systems, Vertex Dental B.V., Soesterberg, The Netherlands) for 15 min at a temperature of 60 °C (Figure 2C,D). Once the polymerization was completed, the supporting structure was removed and finished with a conventional method.

### 2.4. Bonding Procedures for the Bonded Groups

The conventionally and 3D-printed fabricated teeth were positioned in the translucent template (covering the tooth surface except for the area to be bonded) for the surface treatment and bonding standardization. The teeth surfaces were subjected to air-abrasion using alumina particles (50 µm, 10 s, under 0.55 MPa pressure) with a standard 10 mm distance from the tooth surface to the nozzle end [30,31]. The metal brackets were bonded to the printed teeth using the previously described protocol and in accordance with the manufacturer’s instructions.

### 2.5. Three Dimensional-Printed One-Unit Group Preparation

The scanned tooth with the bonded bracket was designed to be printed as one unit (Figure 2A,B). The printing procedures and printing parameters, and the post-curing conditions, performed for the fabrication of the 3D-printed teeth were repeated under the same conditions for the one-unit specimens’ fabrication (Figure 2C,D).

### 2.6. Specimen Storage and Thermal Aging

All the specimens were investigated with a magnification loupe for any voids or defect in the brackets or teeth as well as the bonding area. The specimens were kept in distilled water at a temperature of 37 °C for 24 h. Half of the specimens (*n* = 30, 10/group) were directly tested, while the remaining teeth (*n* = 30, 10/group) were thermally stressed (5000 thermocycling cycles) in a thermal cycling machine (Thermocycler THE-1100-SD Mechatronik GmbH, Feldkirchen-Westerham, Germany). The thermal cycling was performed according to a previous study’s recommendation (5000 cycles, 5 °C and 55 °C, 1 min dwell time, an automatic transfer time of 10 s) [32]

### 2.7. Testing Procedures

The bond strength was evaluated using a universal testing machine (Instron 8871; Instron Co., Norwood, MA, USA). The specimens were fixed to the machine with the knife-edge blade slide parallel to the tooth/bracket interface [26]. A load was applied with 1 mm/min crosshead speed until failure occurred, recording the force in newton (N). To calculate the bond strength (MPa), the forces were divided by the bonded surface area.

### 2.8. Mode of Failure Analysis

After the bond strength test, the failure mode was analyzed per group (cohesive, adhesive or mixed) using a magnification loupe. The adhesive remnant index (ARI) was evaluated for the bonded groups according to Borzangy et al.’s study [33]. For the one-unit printed group, the fracture site (brackets or crown) was used to assess the failure mode.

### 2.9. Statistical Analysis

The collected data were analyzed using SPSS^TM^ version 25.0 (IBM Corp., Chicago, IL, USA). The normality of the data distribution assessment was performed using a Shapiro–Wilk test, which resulted in a normal distribution. The mean and standard deviation were calculated to present the SBS. A one-way ANOVA was conducted to compare the results of the SBS among the groups. Meanwhile, Tukey’s HSD test was performed to allow for pairwise comparisons. All the evaluations were considered statistically significant at a significance level of 0.05.

## 3. Results

Table 1 summarizes the results of the bracket groups. Before and after thermal cycling, the 3D-printed brackets (one-unit) demonstrated the highest SBS value (10.14 ± 1.93 MPa and 8.67 ± 1.53 MPa) when compared with the bonded metal groups (*p* < 0.001), while no statistically significant differences were found between the metal-bonded brackets and the conventional and printed crown groups (*p* > 0.05). Comparing the thermal stress effect for each group, the SBS values of all the groups were significantly decreased, and the 3D-printed brackets (one-unit) recorded the highest SBS value (8.67 ± 1.53 MPa).

Table 2 shows the failure modes between the different tested groups. For the bonded bracket groups, the adhesive failure was the dominant fracture type before and after thermal cycling. In the 3D-printed one-unit group, it was not applicable for the failure mode and only the position of the fractures was described. The fracture was in the brackets, slots and wings with an intact base.

## 4. Discussion

The purpose of the present study was to introduce a new technique for the production of an additively fabricated provisional crown and orthodontic bracket as a single unit. In addition to presenting this new concept, the mechanical strength of the 3D-printed brackets was evaluated both before and after thermocycling. The results demonstrated that the one-unit 3D-printed crown/bracket assemblies exhibited significantly higher bond strength compared to conventionally bonded metal brackets. Based on this findings, the null hypothesis was rejected due to the significant difference between the traditional bonding methods and the proposed one-unit printed restorations.

Three dimensional-printed brackets are considered among the benefits of the recent implementation of 3D printing technology in the orthodontics field [16,34,35]. According to Goracci et al., the many advantages of 3D-printed brackets include the bracket selection, design, modification of the base shape and form, thickness of the base, and size control, in addition to the in-house fabrication, which makes the fabrication process easy and cost-effective [16,34]. Moreover, 3D printing allows for bracket customization and the ability to design appliances. On the other hand, a provisional crown fabricated by the same technique, “additive technology”, was demonstrated with clinically acceptable values. In addition to technology, the material considered to be a crucial factor in the 3D-printed brackets can be fabricated using hybrid ceramic resin or zirconia [10].

Hodecker et al. evaluated the mechanical performance (Martens hardness, indentation modulus, and elastic index) of temporary and permanent 3D resin for orthodontic bracket fabrication and reported that both resins showed comparable results and, at the same time, were superior to commercially available plastic brackets [17]. Additionally, they evaluated the sliding resistance of printed brackets and discovered that 3D-printed brackets and commercially available brackets have comparable sliding resistances [17]. In terms of the mechanical performance of 3D-printed brackets, Papageorgiou et al. [16] indicated the superior mechanical performance of 3D-printed brackets over the contemporary plastic brackets. Beyond their esthetics and high strength, the ability to customize the size and shape of the 3D-printed bracket prior to fabrication represents a significant clinical advantage [16].

Orthodontic treatment success mainly depends on the accuracy of the bracket position when it is positioned and bonded to the tooth properly [36,37,38,39,40]. In the case of brackets bonded to 3D-printed provisional crowns that were fabricated, the bonding issue might remain a drawback, which negatively affects the treatment [30,41]. To leverage adaptive technology and reduce dependency, this study proposed the concept of a one-unit crown and bracket.

In the current study, the SBS values of the bonded groups fell below the clinically acceptable threshold both before and after thermocycling. This outcome may be attributed to the previously discussed limitations of the bonding mechanism as well as to the adverse effects of thermal stress at the bracket/crown interface [1,30]. In contrast, the one-unit 3D-printed groups demonstrated bond strength values exceeding clinical recommendations, even following thermocycling. These findings confirm the durability and superior mechanical performance of the one-unit restorations, primarily due to their integrated design, which maintained acceptable strength levels even before thermal aging.

Additionally, the results showed the high strength of the 3D-printed one-unit groups when compared with the bonded groups. In the bonded groups, the adhesive failure type was dominant. Notably, adhesive failures remained the most prevalent fracture type, despite the application of standardized surface treatment across all the bonded specimens. This finding is in agreement with Shahin et al.’s study [30], while it is in disagreement with Çokakoğlu et al. [26]. Çokakoğlu et al. [26] bonded a 3D-printed bracket to a printable permanent crown and reported that 80% of the fractures were cohesive fractures within the bracket accompanied by bracket fractures.

This limitation could be addressed by utilizing different printing technologies and adjusting the printing parameters, such as altering the printing orientation, printing layer thickness, and post-polymerization conditions [34,42,43,44]. Additionally, the printing parameters have a crucial impact, as reported in a recent study [26], which investigated the effect of different printing parameters on the shear bond strength of 3D-printed brackets bonded with a printable permanent crown.

It was reported that the printing orientations significantly affect the strength of printed resins [34,45]. By changing the printing orientations, the direction of the load to the printing layer direction is changed and could affect the strength of the printed object [45,46]. The printing orientation influences the alignment of the layers relative to the direction of the applied load. Regarding the printing parameters’ effect, Çokakoğlu et al. [26] compared different printing orientations (0, 45, and 90 orientations) and found that the printing orientations’ affect the strength and fracture resistance of 3D-printed brackets, and the least amount of fractures was found with the 90-orientation printed bracket. In Çokakoğlu et al.’s [26] study, 45-degrees showed the lowest strength with more adhesive failure. They attributed this to the support structure’s position and its removal after printing. Çokakoğlu et al. [26] found that when the bracket was printed with 45 orientations, the bracket base design was affected during removal. In the present study, the 0-orientaion approach was used according to the manufacturer’s recommendation. Also, the design of the printed one-unit technique overcame the drawbacks of the brined bracket alone in terms of the support structure position and removal to minimize the risk of bracket base damage. However, the printing orientations play an important role in the one-unit technique, which necessitates further investigations with different printing directions according to the printed axes (x, y, and z), which might affect the strength and accuracy of the printed object [47]. Therefore, by considering the direction of the traction forces, the layer orientation at the bracket/crown interface can be optimized to enhance the mechanical properties [46].

Additional factors, such as the printing layer thickness, might be considered as influencing factors in relation to the strength and accuracy of 3D-printed resins [48,49]. Different layer thicknesses were claimed according to the manufacturer, with the most common thicknesses ranging from 25 µm to 100 µm [34,45]. The printing layer thickness mainly affects the number of layers per printed object and the efficacy of the polymerization of each layer. This would be reflected in the dimensional accuracy as well as the bonding between layers and might reduce the strength [34,50]. However, the reduced printing layer thickness was recommended due to the efficacy of the polymerization and light penetrations through thin layers based on the recent review conducted by Gad et al. [45]. Therefore, in the present study, the thickness was standardized to 50 µm according to the manufacturer’s recommendation and the layer thickness used in a previous study [26]. Considering the printed brackets’ size with high strength and appropriate accuracy in comparison to other printed objects, decreasing the layer thickness could be suggested in further investigations. This aligns with [50], which compared a wide range of layer thicknesses (25 µm, 50 µm, 100 µm, and 175 µm) on the printed model and reported that decreasing the printing layer thickness up to 25 µm resulted in the highest accuracy. However, decreasing the layer thickness resulted in a longer printing time and may extend longer to be four times that of 100 µm [49], which may affect the in-house printing technology owing to being more time-consuming. Accordingly, clinicians are advised to make informed decisions about the optimal layer thickness by considering the workflow dynamics, procedural time constraints, and other practical factors relevant to their specific clinical sittings.

The post-polymerization conditions also play a critical role in determining the final strength of printed resins [51]. The printed resin was called green state as the polymerization was not completed [34]. Therefore, further polymerization processes with different printing conditions resulted in specimens with optimum strength compared to the green state materials [26]. The post-polymerization minimizes the risk of deformation and shrinkage of resin-based material [52]. There are many controlling factors affecting the post-polymerization process, including the cleaning solution, the curing unit and the conditions of specimens within the curing unit, curing time, curing conditions, and curing temperature [34]. During the post-polymerization process, within the curing unit, it was recommended to submerge the specimens in inert mediums, such as nitrogen gas or a glycerin bath, to prevent the formation of an oxygen inhibition layer [53,54]. The importance of the glycerin medium was proved by Çokakoğlu et al. [26], who reported that the brackets processed without the glycerin medium affected the binding strength in terms of adhesive failure occurrences. Owing to the crucial effect of the post-polymerization impact on the strength of printed brackets, it could be recommended to investigate one-unit technique performances when treated with different post-printing parameters (rinsing solution, curing units, curing time, and/or curing temperature as well as the combination of different parameters), as categorized in the recent review [34].

In the one-unit 3D-printed groups, the fracture was in the brackets, where the most common fracture sites were in brackets’ wings and slot areas, which are considered weak points in the 3D brackets’ design. The fracture may be attributed to the low strength of the printed specimens, which could be related to the printing nature being layer-by-layer [34,55]. This could be overcome based on this study’s recommendations for further investigation with different printing technologies, newly introduced materials and combinations of printing parameters. By changing the parameters, the layer directions will be changed and could modify the characteristics of the printed object. This finding directed the research toward different printing parameters and conditions, as well as printing material types and printing technologies, aiming to improve the strength of the printed resins [16,26,45]. In this way, the risk of slot and wing damage could be reduced and the limitation of bracket fracture for future and clinical applicability could be overcome. An additional challenge that could be considered with fractured brackets is tissue irritation, especially when the fracture exhibited a sharp surface that could affect the tissue. Improving the mechanical performance of printed brackets through the suggested modalities might decrease the incidence of bracket fracture; however, this should be considered in future studies or with the fabrication of an occlusal template for tissue protection to be placed over the teeth by the patient with same incidence or instruct the patient to use wax to cover the fractured wing.

Clinically, this technique proves valuable when utilizing 3D printing technology instead of printing provisional restorations and brackets separately, or using metal or ceramic brackets, then bonding to the crown. In addition to the advantageous of permitting adjustments of the brackets’ design and position with digital workflow. Additionally, this technique can be seamlessly integrated into a digital workflow for orthodontic treatment. Besides reducing the chair-side time, this technique facilitates accurate bracket placement in alignment with the treatment plan and digital design, thereby minimizing the errors that occur with conventional bonding methods [40,56]. Clinical applicability remains an area for further investigation as the bracket slot of the one-unit 3D-printed bracket and crown is made of resin material, which could affect the friction of the wire as it slides through the bracket slot.

The introduced technique presented some limitations, such as using one printed resin and one printing technology, which was considered a limitation of the present study. In addition to the in vitro study limitations, the failure to simulate the complex environment of the oral cavity, such as the masticatory forces, salivary enzymes, and biofilm formation, might affect the generalizability and clinical relevance of the findings. Therefore, future studies conducted with different printing technologies and new 3D-printed resins (provisional or permanent) and involving testing in conditions simulating the oral environment are recommended.

## 5. Conclusions

The 3D-printed one-unit provisional restoration with brackets showed high strength compared with the conventional bonded groups. Thermal cycling impacted the bond strength of both the 3D-printed one-unit restorations and the conventionally bonded groups. The one-unit 3D-printed restoration approach is recommended as a viable option for provisional restorations. To verify the technique and assess its clinical implications, further investigations using different resins with different printing parameters are recommended. Moreover, in vivo studies simulating oral conditions are essential to validate the clinical applicability.

## Figures and Tables

**Table 1:** Results of the shear bond strength (MPa) for all the tested groups.

	CCMBB	3DPCMBB	3DPCB-One-Unit	
Thermal stress	Conventional crown and metal-bonded bracket Mean ± SD	3D-printed crown and metal-bonded bracket Mean ± SD	3D-printed crown and bracket (one-unit) Mean ± SD	*p*
Before	5.67 ± 1.02 ^a^	5.07 ± 0.092 ^a^	10.14 ± 1.93	*p* < 0.001 *
After	3.86 ± 0.99 ^a^	3.66 ± 1.03 ^a^	8.67 ± 1.53	*p* < 0.001 *
*p*	0.009 *	0.013 *	0.041 *	

* *p* < 0.05 statistically significant. Same small letter horizontally indicates nonsignificant differences.

**Table 2:** Mode of failure. The ARI score of the printed bracket groups and the position of failure in the one-unit printed groups.

	Mode of Failure	Conventional Crown and Bonded Bracket	3D-Printed Crown and Bonded Bracket	3D-Printed Crown and Brackets (One-Unit)
No TC	Adhesive (Score 0)	7	8	All fractures in printed brackets
	Cohesive (Score 1)	-	-	
	Mixed (Score 2)	3	2	
	(Score 3)			
After TC	Adhesive (Score 0)	9	10	
	Cohesive (Score 1)	-	-	
	Mixed (Score 2)	1	-	
	(Score 3)			

Score 0, no adhesive left on the crown surface. Score 1, less than half of the adhesive left. Score 2, more than half of the adhesive left. Score 3, all the adhesive left on the crown surface, with a distinct impression of the bracket mesh.

## Data Availability

The original contributions presented in the study are included in the article, further inquiries can be directed to the corresponding author.
